# Insight into chromatin compaction and spatial organization in rice interphase nuclei

**DOI:** 10.3389/fpls.2024.1358760

**Published:** 2024-05-28

**Authors:** Alžběta Doležalová, Denisa Beránková, Veronika Koláčková, Eva Hřibová

**Affiliations:** Institute of Experimental Botany of the Czech Academy of Science, Centre of Plants Structural and Functional Genomics, Olomouc, Czechia

**Keywords:** 3D immuno-FISH, chromosome painting, chromosome territory, rice, spatial organization, microscopy

## Abstract

Chromatin organization and its interactions are essential for biological processes, such as DNA repair, transcription, and DNA replication. Detailed cytogenetics data on chromatin conformation, and the arrangement and mutual positioning of chromosome territories in interphase nuclei are still widely missing in plants. In this study, level of chromatin condensation in interphase nuclei of rice (*Oryza sativa*) and the distribution of chromosome territories (CTs) were analyzed. Super-resolution, stimulated emission depletion (STED) microscopy showed different levels of chromatin condensation in leaf and root interphase nuclei. 3D immuno-FISH experiments with painting probes specific to chromosomes 9 and 2 were conducted to investigate their spatial distribution in root and leaf nuclei. Six different configurations of chromosome territories, including their complete association, weak association, and complete separation, were observed in root meristematic nuclei, and four configurations were observed in leaf nuclei. The volume of CTs and frequency of their association varied between the tissue types. The frequency of association of CTs specific to chromosome 9, containing NOR region, is also affected by the activity of the 45S rDNA locus. Our data suggested that the arrangement of chromosomes in the nucleus is connected with the position and the size of the nucleolus.

## Introduction

Nuclear DNA is condensed together with structural proteins into higher-order chromatin structures, which serve as substrates for important biological processes, such as DNA replication, transcription, and genome repair (Misteli, 2020). While the chromatin is packed into visible, highly condensed chromosome structures during mitosis, the chromatin decondensation in the interphase of the cell cycle prevents from recognizing the borders of the individual chromosomes. This evokes fundamental questions on how the chromatin is packed into chromosomes, how the chromosomes are organized during the interphase of the cell cycle, and how the chromatin packing and chromosome positioning influence the biological processes.

The organization of chromatin during the interphase can be analyzed by two methodological approaches: by the high-throughput chromosome conformation capture (Hi-C) technique, followed by polymer modeling (Lieberman-Aiden et al., 2009; Giorgetti et al., 2014; Gibcus et al., 2018), and by the three-dimensional fluorescence *in situ* hybridization (3D-FISH) and microscopic techniques (Bass et al., 1997; Markaki et al., 2012). The Hi-C method combines 3C technique (Dekker et al., 2002) and next-generation sequencing (Lieberman-Aiden et al., 2009) to examine contact/interaction frequencies between chromosomal regions. Recently, Hi-C techniques have been used in many living organisms to describe chromosome contact patterns, genome packing, and 3D chromatin architecture at much higher resolution (tens to hundreds of kilobases) than it was provided by 3D-FISH (Grob et al., 2014; Dong et al., 2018; Dumur et al., 2019; Concia et al., 2020; Golicz et al., 2020). However, the majority of Hi-C studies in plant species were performed on pooled tissues and thus could not provide information on the variability in the spatial organization of individual chromosomes in 3D space of the interphase nuclei (Wang et al., 2015; Dong et al., 2017; Concia et al., 2020). This information can be acquired by the application of recently developed cytogenetic techniques, oligo-painting, and 3D-FISH, which enable to visualize individual genome regions in 3D space of nuclei (Howe et al., 2013; Han et al., 2015).

Hi-C studies in metazoans and mammals revealed the existence of megabase-long chromatin compartments containing either active and open chromatin (A compartments), or inactive and closed chromatin (B compartments). Hi-C also allowed to describe the organization into smaller, self-interacting topologically associated domains (TADs), regulatory landscapes of chromosomes, in the animal interphase nuclei (e.g. Sexton and Cavalli, 2015; Ramírez et al., 2018; Szabo et al., 2018). Finally, it was showed that genes belonging to the same TADs display similar expression dynamics, suggesting that their physical association is functionally related to gene expression control (de Graaf and van Steensel, 2013). In plants, 3D chromatin architecture is different. For instance, TADs were not observed in *A. thaliana* (Feng et al., 2014; Wang et al., 2015; Liu et al., 2017), instead, their presence seems to be linked to species with larger genomes (Dong et al., 2017; Liu et al., 2017; Concia et al., 2020; Golicz et al., 2020). Since TADs have not been recognized in all plant species, their role in the dynamics of plant chromatin remains unclear.

Complementary cytogenetic data to Hi-C studies are still missing in larger set of plants, only few of them were published in Arabidopsis (Feng et al., 2014; Grob et al., 2014). In plant research, chromosome distribution in interphase nuclei was studied by FISH with probes specific to the functional chromosome domains, such as centromeres and telomeres (Avivi and Feldman, 1980; Anamthawat-Jónsson and Heslop-Harrison, 1990; Schwarzacher and Heslop-Harrison, 1991; Rawlins et al., 1991; Montijn et al., 1994; Hou et al., 2018; Liu et al., 2020). These studies confirm the first microscopic observations made by Carl Rabl, who predicted that the chromosome positioning in interphase nuclei follows their orientation in the preceding mitosis (Rabl, 1885; reviewed by Cremer et al., 2006). The so-called Rabl configuration, with centromeres and telomeres oriented on opposite poles of nuclei, was originally assigned to plants with large genomes. The concept of a Rabl-like organization in plant species with large genomes and non-Rabl organization of interphase chromosomes in plants with small and medium genomes has been disproved early after it was proposed (Dong and Jiang, 1998; Fujimoto et al., 2005). In rice, the majority of nuclei in somatic cells lack Rabl configuration (Prieto et al., 2004; Santos and Shaw, 2004; Němečková et al., 2020), however, chromosomes of pre-meiotic cells in anthers or xylem-vessel precursor cells seem to assume the Rabl configuration (Prieto et al., 2004; Santos and Shaw, 2004). Compared to numerous studies on the centromere-telomere organization in plant interphase nuclei (Fujimoto et al., 2005; Idziak et al., 2015; Němečková et al., 2020; Shan et al., 2021; Nowicka et al., 2023), the visualization of the spatial positioning of individual chromosomes during interphase stays widely unknown.

Dynamics of interphase chromosomes was studied in inter-specific and inter-generic hybrids by the visualization of parental subgenomes using the genomic *in situ* hybridization (GISH) (Leitch et al., 1990). The use of specific cytogenetic lines (addition lines, translocation lines) then enabled to study the positioning of the introgressed chromosomes in 3D space of cell nuclei, representing various stages of the cell cycle, as well as meiotic cells (Aragón-Alcaide et al., 1997; Abranches et al., 1998; Koláčková et al., 2019; Perničková et al., 2019). The mutual position of individual chromosomes during interphase was studied in *Arabidopsis thaliana* using the BAC pools-based chromosome painting technique, showing that individual chromosomes tend to occupy the separated territories (Pečinka et al., 2004). The extremely small genome of Arabidopsis is characterized by a specific, rosette-like chromosome configuration (Fransz et al., 2002), which was not observed in any other plant species, thus we can not expect that the chromosome organization and dynamics revealed in *Arabidopsis* is universal to other plant species. Robaszkiewicz et al. (2016) later analyzed the chromosome positioning in 3D space of *Brachypodium distachyon*, which possesses Rabl organization, and provided the first insight into the large variability of the interphase chromosome organization. However, the high level of variability in mutual chromosome organization shown in the study could have been caused by the use of nuclei isolated from the pooled root tissue (Robaszkiewicz et al., 2016).

Advanced techniques of optical microscopy, so called super-resolution microscopy, enable to study objects at resolutions higher than those limited by the diffraction limit of the light (Abbe, 1873). Out of them, fluorescence nanoscopy methods expanded optical imaging to reach the nanometer resolution range (Sahl et al., 2017). In our study, we used STED nanoscopy which can reach xy-resolution less than 60 nm, and enables the acquisition of three-dimensional images (Dumur et al., 2019; Moors et al., 2021; Frolikova et al., 2023). To provide information on chromatin compaction during the interphase of the cell cycle, mild formaldehyde fixation of the nuclei and their further mounting in polyacrylamide gel was used to preserve 3D chromatin structure and to avoid chromatin destruction during the sample preparation (Howe et al., 2013; Bass et al., 2014; Němečková et al., 2020).

Our present study provides the first insight into chromatin compaction and variability of the spatial organization of CTs during the interphase of the cell cycle in highly dynamic root meristematic cells and diversified leaf nuclei. The use of super-resolution STED microscopy revealed different levels of chromatin compaction in root and leaf nuclei. Different types of mutual CTs positioning, which varied between the root and leaf interphase nuclei, were observed using 3D immuno-FISH experiments with chromosome-specific painting probes.

## Materials and methods

### Plant material, seeds germination and sample preparation

Seeds of rice cultivar Nipponbare (*Oryza sativa*, 2n=2x=24) were obtained from Prof. Ohmido Nobuko, Kobe University, Japan. Seeds were soaked in distilled water and aerated for 24 h. After that, seeds germinated in a biological incubator at 24°C on moistened filter paper in a Petri dish until the primary roots were 3–4 cm long. Young leaves were collected 10 days after germination. Suspension of intact nuclei was prepared according to Doležel et al. (1992). Briefly, root tips or leaves (without leaf base) were cut and fixed with 2% (v/v) formaldehyde in Tris buffer (10 mM Tris, 10 mM Na2EDTA, 100 mM NaCl, 0.1% Triton X-100, 2% formaldehyde, pH 7.5) at 4°C for 30 min and washed three times with Tris buffer at 4°C. Meristematic parts of root tips (~1 mm long) were excised from 70 roots per sample. Root meristems were homogenized in 500 µl LB01 buffer (Doležel et al., 1989) by Polytron PT 1200 homogenizer (Kinematica AG, Littua, Switzerland) for 13 s at 14 500 rpm. Leaves were chopped by razor blade. Finally, the suspensions were filtered through a 20 µm nylon mesh and analyzed using the FASCAria II SORP flow cytometer and sorter (BD Bioscience, San Jose, USA). Nuclei representing the G1 phase of the cell cycle were sorted into 1x meiocyte buffer (Bass et al., 1997; Howe et al., 2013).

### Root microtome sectioning and fluorescence *in situ* hybridization

Roots fixed with 2% (v/v) formaldehyde in Tris were embedded in Cryo-Gel (Leica Biosystems, ID:39475237) and cut into 20 µm thick sections using a cryostat (Leica CM1950). The resulting segments were transferred to super-frost slide (Thermo Scientific) and allowed to dry overnight at room temperature. Prior to FISH, slides with root segments in cryo-gel were washed in 1x PBS for 10 minutes, and subsequently dehydrated in the ethanol series (70%, 85%, 100% ethanol), each for 2 minutes. Hybridization mix (50 µl) containing 50% (v/v) formamide, 10% (w/v) dextran sulfate in 2x SSC, 1 µg sheared salmon sperm DNA (Invitrogen, AM9680) and 200 ng probe was added onto the slides and denatured for 8 min at 78°C, followed by slow cooling process (50°C 1 min, 45°C 1 min, 40°C 1 min, 38°C 5 min). Slides were then hybridized overnight at 37°C. Next day, slides were washed in 4xSSC three times for 5 minutes, and root sections were counterstained with DAPI in VECTASHIELD Antifade Mounting Medium (Vector Laboratories, Burlingame, CA, USA).

### Probes for FISH

Oligonucleotides specific for individual chromosomes were identified in the reference genome sequence of *Oryza sativa* cv. Nipponbare (version_7.0; http://rice.uga.edu/; Kawahara et al., 2013) using the Chorus v2 program pipeline (Zhang et al., 2021). Two sets of oligomers were synthesized by Daicel Arbor Biosciences (Ann Arbor, Michigan, USA). Labeled oligomer probes were prepared according to Han et al. (2015). Probes specific for the long and short arms of chromosome 2 were labeled by biotin-16-dUTP and by aminoallyl-dUTP-CY3, and chromosome 9 was labeled by digoxigenin-11-dUPT and aminoallyl-dUTP-CY5 (Jena Biosciences, Jena, Germany). The painting probe of longer chromosome 2 contained 40,000 unique 45-mers and the painting probe specific to short chromosome 9 contained 20,000 unique 45-mers. Probe specific for 45S ribosomal DNA was amplified using specific primers (Ohmido and Fukui, 1995) and directly labeled with aminoallyl-dUTP-CY5 (Jena Biosciences, Jena, Germany). Chromosomes 2 and 9 were selected based on the previous study of Dong et al. (2018), which proposed the presence of two sets of chromosomes differing in level of their association based on the Hi-C results. Long, sub-metacentric chromosome 2 (member of chromosome set which showed close association), and short acrocentric chromosome 9 containing NOR region and belonging to the set of chromosomes which did not show apparent association (Dong et al., 2018). Prepared probes were confirmed by FISH on standard chromosomes spreads prepared according to (Hou et al., 2018).

### Immuno-staining and fluorescence *in situ* hybridization

Flow sorted nuclei were mounted in polyacrylamide gel according to Němečková et al. (2020). To visualize 45S rDNA, chromosome 2 and chromosome 9, and fibrillarin, staining procedures and washes were performed according to Němečková et al. (2020). Primary antibody anti-fibrillarin was diluted at 1:100 (ab4566, Abcam, Cambridge, UK). The hybridization mix for FISH contained 400 ng of individual probes.

### Sample preparation for STED microscopy

Flow sorted nuclei were mounted in polyacrylamide gel onto silane-cover glass. High-precision cover glasses were prepared according to de Almeida Engler (2001) with some modifications. The slides were washed in distilled water for 15 min, then in ethanol for 30 min, and let air dry for 10 min at room temperature (RT)Slides were placed into Petri dish and soaked in freshly prepared 2% 3-aminopropyltriethoxysilane (Sigma) in acetone for 30 min at RT by shaking. the slides were then washed twice in distilled water, dried overnight at 37°C, and stored at RT. After gel polymerization, polyacrylamide pads were washed in MBA buffer (Howe et al., 2013; Bass et al., 2014) and left to dry at RT. Glycerol mounting medium AD-MOUNT S (ADVi, Říčany, Czech Republic) with SPY650-DNA (diluted 1:1000) (Spirochrome AG, cat#: SC501, Stein am Rhein, Switzerland) was applied onto the pads and covered with a microscopic slide.

### Confocal and STED microscopy, and image analysis

**Figure 1:**
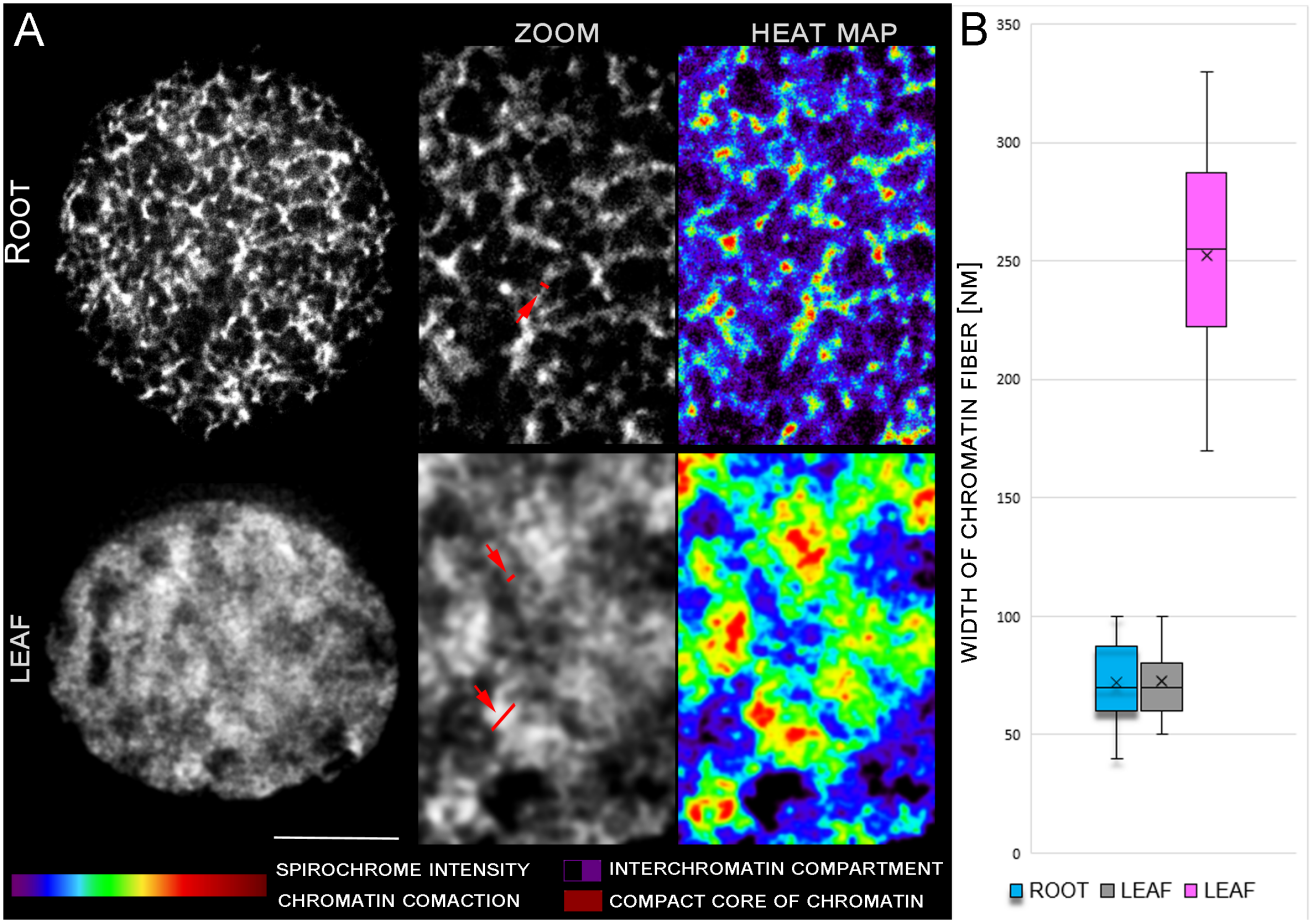
STED analysis of chromatin condensation in G1 nuclei of young leaves and root meristem. DNA was stained by spirochrome (white). **(A)** Differences in DNA structure are clearly visible in zoomed pictures and heat maps. Red arrows indicate region of chromatin width measurement. **(B)** Graph of discrete chromatin fiber measurements. Leaf fibers measurements displayed two different groups of chromatin size (grey and pink plot) Scale bar: 2 µm.

Images were acquired using Leica TCS SP8 STED 3X confocal microscope (Leica Microsystems, Wetzlar, Germany) equipped with 63x/1.4 NA Oil Plan Apochromat objective and Leica LAS-X software with Leica Lightning module. Image stacks were captured separately for each chromosome using 647 nm, 561 nm, 488, and 405 nm laser lines for excitation and appropriate emission filters. Typically, an image stack of about 50 slides with 0.15 µm spacing was acquired. Root sections were acquired via the Navigator module using a 63x objective and the final picture was created by the Mosaic merge function. Chromatin structure of leaf and root nuclei was captured in the STED mode with 100x 1.4 NA STED oil objective. The pinhole was set to 0.75 AU. The resolution was estimated using LAS-X software, according to full width at half maximum criterion (FWHM). Chromatin signal labeled by spirochrome (SPY650-DNA) was captured with a lateral resolution of approximately 52 nm. LAS-X software was also used to produce color heat maps of individual nuclei. The size of chromatin was measured in Leica software. Measurements of visible chromatin fibers were taken as displayed in the **Figure 1** and **Supplementary Figure 1**. Size of chromatin fibers was obtained by random measurement of 50 different locations in the root nuclei space, and 100 positions were measured in leaf nuclei (**Supplementary Figure 1**).

3D models of microscopic images and volume calculations were performed using Imaris 9.7 software (Bitplane, Oxford Instruments, Zurich, Switzerland). The volume of each nucleus, nucleolus, and chromosome territories was estimated based on the primary intensity of fluorescence obtained by microscopy (e.g. Koláčková et al., 2019; Perničková et al., 2019; Randal et al., 2022). If two separated territories, corresponding to two homologs of the analyzed chromosomes were observed after 3D-FISH, Imaris software calculated the volume of individual territories, and the mean volume of the chromosome was calculated as a sum of both territories. Imaris function ‘Surface’ was used for modeling the chromosome arrangement in the nucleus and for modeling the 45S rDNA, chromosomes, and fibrillarin. To create volumes of nucleus, nucleolus, chromosomes and 45s rDNA, surface detail 200 nm was used and background subtraction was set to the diameter of the measured object (5 µm for nucleus, 2 µm for individual chromosomes, and 0.5 µm for 45s rDNA). Shells of nucleus were created to be showed as surface of nucleus (DAPI) with different diameter of background. Chanel contrast was adjusted using ‘Chanel Adjustment’ tool, and videos were created using the ‘Animation’ function. Approximately 100 nuclei were analyzed for each selected variant.

## Results

### Variation in chromatin condensation and nuclei features

To analyze and compare the levels of chromatin condensation in G1 interphase nuclei of young leaves and root meristems, we applied the stimulated emission depletion (STED) microscopy. Mildly fixed flow sorted G1 nuclei from leaves and root meristems were mounted in polyacrylamide gel onto silane-coated high-precision cover glass to preserve their 3D structure. STED analysis uncovered the detailed chromatin ultrastructure and revealed the differences in the levels of chromatin compaction between G1 nuclei isolated from leaves and root meristems. G1 nuclei of the root meristem, which underwent repeated and rapid cell division, were characterized by more relaxed chromatin and apparent ultra-structures (**Figure 1A**). In comparison, a more compact structure of chromatin and presence of lower amount of interchromatin compartments was found in G1 nuclei isolated from differentiated leaf cells (**Figure 1A**). Chromatin condensation in G1 nuclei isolated from both tissues were also visualized as color heat maps (e.g. Cremer et al., 2017; Cremer and Cremer, 2019), which display the differences in the general chromatin organization between the root meristem and leaf G1 nuclei (**Figure 1A**). The width of distinguishable chromatin fiber in G1 nuclei reach the same value 72 nm in root and leaf tissue. In leaf nuclei, denser chromatin structures with diameter 252 nm were observed (**Figure 1B**).

**Figure 2:**
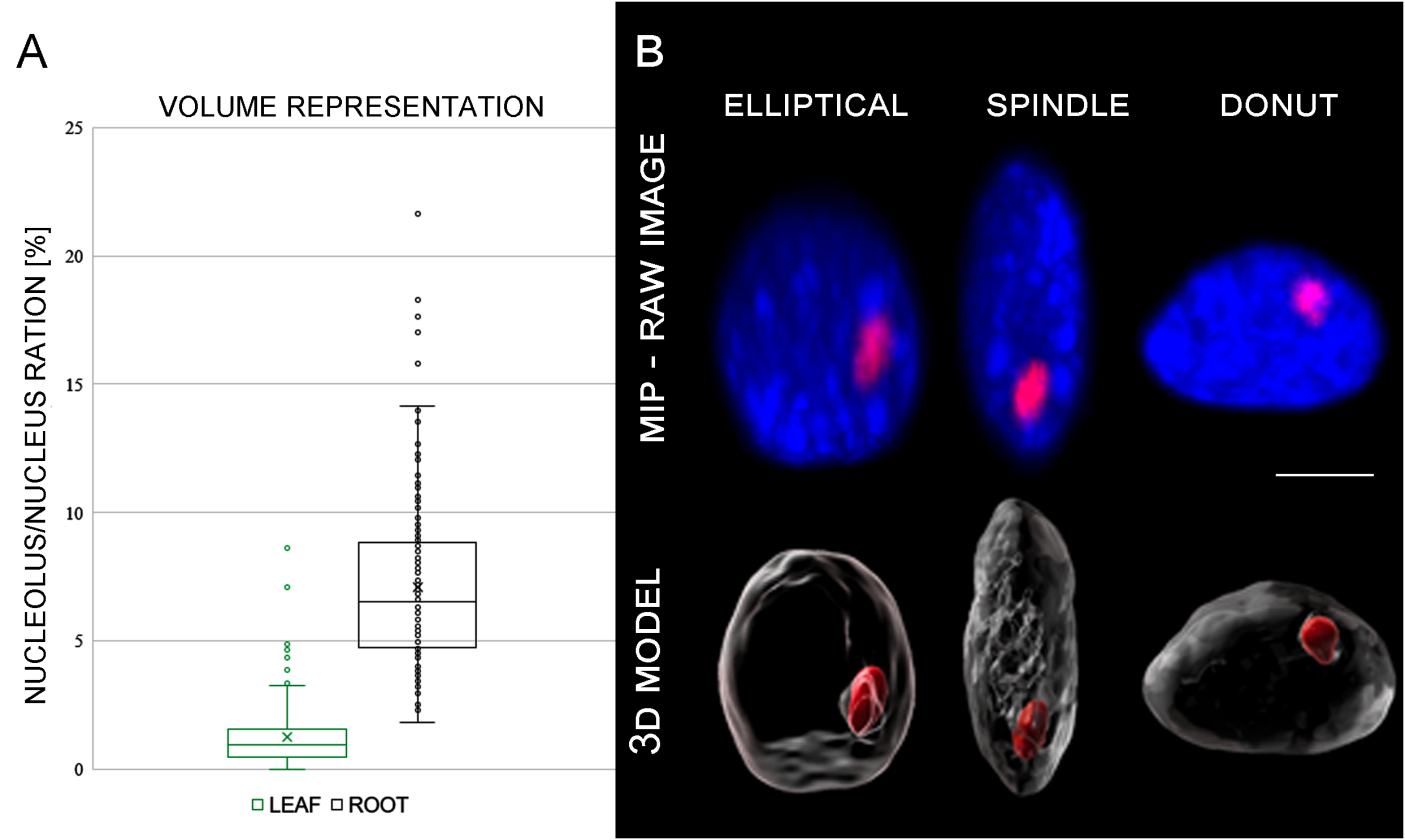
**(A)** Graph representing average nucleolus/nucleus ration of each individual cell. **(B)** Differences in shape of the analyzed nuclei. Maximal intensity projection (MIP) of nuclear DNA stained with DAPI (blue). Nucleolus was visualized using fibrillarin immunolabeling (red).

Likewise, the nuclei volume of G1 nuclei isolated from the root meristematic zones was more than three times higher (199 µm^3^) compared to leaf nuclei (59.6 µm^3^) (**Table 1**). Similar situation was observed also for nucleoli. Here we have to emphasize, that the nucleoli were determined by immunodetection with nucleolus-specific protein fibrillarin that locates to the dense fibrilarin components (DFC) region of nucleolus representing up to 70% of the nucleoli (Brown and Shaw, 1998; Dvořáčková and Fajkus, 2018). The volume specific to DFC region represented 14 µm^3^ (7.1% of the root nucleus) on average, and 0.7 µm^3^ (1.2%) of the leaf nucleus (**Table 1**; **Figure 2A**; **Supplementary Video 2**).

**Table 1:** Analysis of all tested G1 nuclei specific for both analyzed tissues.

	Volume of nucleus [µm3]	Volume of nucleolus [µm3]	Number of G1 nuclei	Mean diameter of nucleus [µm]			Shape of nucleus [%]		
				x	y	z	Elliptical	Spindle	Flattened
Root	199.01± 50.75	14.13 ± 4.67	189	7.14	6.62	8.95	66.67	20.63	12.70
Leaf	59.57 ± 23.85	0.74 ± 0.24	185	4.53	4.23	5.62	65.41	20.54	14.05

The variation in shape and volume of nucleus and nucleolus is shown.

Further analyses of almost 200 G1 nuclei specific for both analyzed tissues revealed variation in their shapes (**Figure 2B**). Majority of the nuclei had elliptical shape (~ 67%), and the rest of the G1 nuclei had spindle-like (~ 21%) and flattened (~ 13%) shapes in the root meristematic cells. Proportion of the G1 nuclei shapes was almost identical for both studied tissues (**Table 1**; **Figure 2B**).

### Mutual position of chromosomes in G1 interphase nuclei

**Figure 3:**
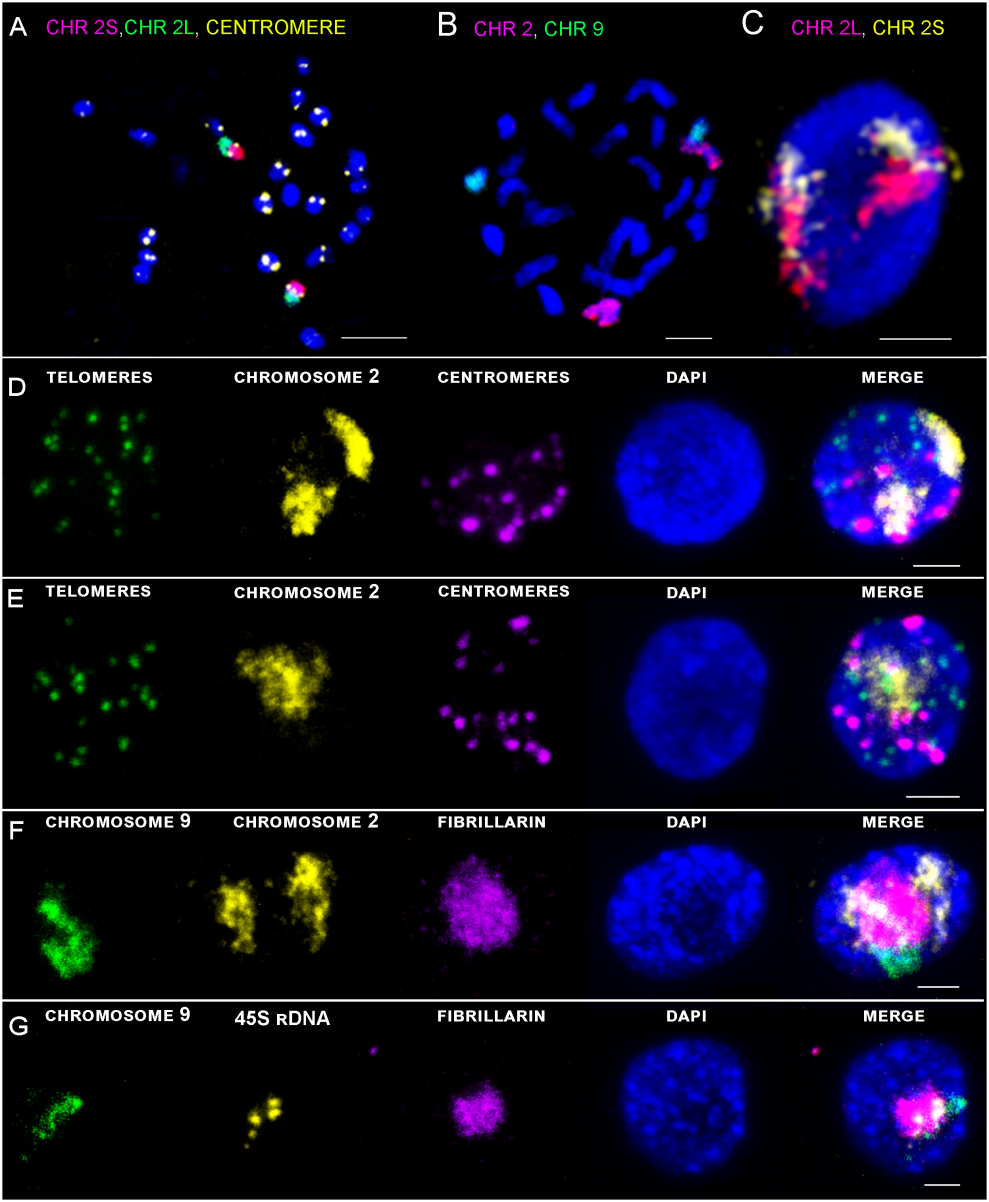
Maximal intensity projection of confocal scanning of chromosomes and G1 nuclei of rice after immuno-FISH localization on flow sorted G1 nuclei of root meristem. **(A)** Visualization of centromere (yellow), short arm of chromosome 2 (2S) (pink), and long arm (2L) (green) on metaphase chromosomes. **(B)** Visualization of chromosome 2 (pink) and chromosome 9 (green) by oligo-painting FISH on prometaphase chromosomes. **(C)** Visualization of two separate chromosome territories corresponding to two homologous chromosomes. Long arm of chromosome 2 in pink, short arm of chromosome 2 in yellow. **(D, E)** DNA was counterstained with DAPI (blue). Scale bar: 3 µm. Immuno-FISH localization of specific probes on G1 nuclei of root meristem **(D, F, G)** and leaf tissue **(E)**. DNA was counterstained with DAPI (blue). Scale bar: 2 µm.

Detailed positioning of two chromosomes was analyzed thanks to the two specific painting probes for long sub-metacentric chromosome 2, and short acrocentric chromosome 9 containing NOR region. Sensitivity and suitability of the painting probe for chromosome identification *in situ* was confirmed by FISH on standard chromosomes spreads (**Figures 3A–C**), and further on flow sorted G1 nuclei of root meristem (**Figures 3D–F**) and leaf tissue (**Figure 3G**).

**Figure 4:**
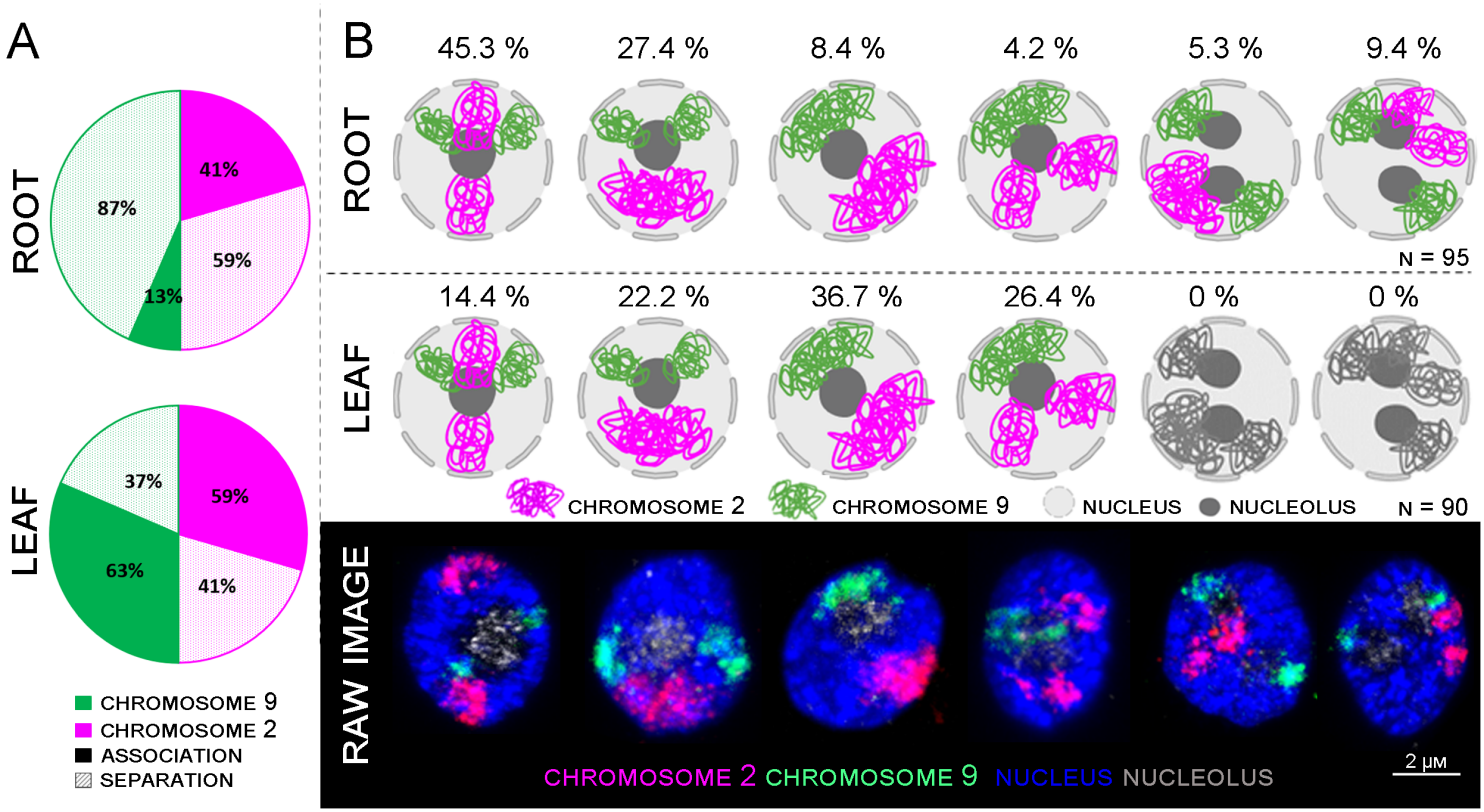
Comparison between root and leaf chromosome arrangements. **(A)** Graph summarizing the chromosome associations in root and leaf tissues displayed in **(B)**. **(B)** Models of individual arrangements created with BioRender.com, based on raw data observation captured by confocal microscopy.

3D-FISH with the chromosome painting probes on G1 nuclei of rice revealed the presence of specific regions in both examined tissue types and confirmed the presence of chromosome territories (CTs), which were predicted by Hi-C studies (Dong et al., 2018). Painting FISH demonstrated variability in constitution of the CTs, which were present either as two separated territories corresponding to two homologous chromosomes in G1 nuclei (**Figures 3C, D, F**), or as one large territory in which homologous chromosomes were tightly connected (**Figure 3E**). In general, higher proportion of G1 nuclei isolated from the leave tissue showed close association of homologous chromosomes visualized as one large CT (63% for chromosome 9; and 59% for chromosome 2), compared to root meristem, which predominantly contained G1 nuclei with two separated CTs (87% for chromosome 9; and 59% for chromosome 2) (**Figure 4A**). The comparison of the volumes of both territories corresponding to homologous chromosomes did not reveal significant differences. Chromosome territory 1 and 2 of chromosome 2 represented 8,23% and 8,16% of the nucleus volume in root meristematic cells and 8,76% and 8,76% in leaf cells. The volume of associated territory of two homologous CTs was equal to the sum of two separate homologous CTs (**Table 2**). Interestingly, chromosome territory 1 and 2 of chromosome 9 covered slightly higher volume in leaf (8,31% and 7,72%) compare to root (5,39% and 5,49%) (**Table 2**).

**Table 2:** Characteristics of analyzed G1 nuclei and chromosome territories CTs.

	Number of analyzed G1 nuclei	Mean volume of nucleus [µm^3^]	Mean volume of separated CTs of chromosome 2 [µm^3^]	Mean volume of associated CTs of chromosome 2 [µm^3^]
**Root**	95	198.94 ± 55.34 100%	16.38 ± 3.68 8.23%	16.23 ± 5.16 8.16%
**Leaf**	90	60.42 ± 22.23 100%	5.29 ± 0.90 8.76%	5.29 ± 2.09 8.76%

	Number of analyzed G1 nuclei	Mean volume of nucleus [µm^3^]	Mean volume of separated CTs of chromosome 9 [µm^3^]	Mean volume of associated CTs of chromosome 9 [µm^3^]
**Root**	189	199.01 ± 50.75 100%	10.73 ± 2.07 5.39%	10.94 ± 2.69 5.49%
**Leaf**	185	59.57 ± 23.85 100%	4.95 ± 1.35 8.31%	4.60 ± 1.87 7.72%

Six different patterns of mutual arrangement of the two analyzed chromosomes in G1 nuclei of root meristem were observed (**Figure 4B**). Approximately 45,3% of the examined root meristematic nuclei contained chromosomes 2 and 9 organized in two separate CTs concurrently, and additional 27,4% of the nuclei contained chromosome 9 arranged in two separate CTs, and chromosome 2 in one large CT. The rest of analyzed nuclei (14,7%) contained two nucleoli, and in all these cases, CTs of chromosome 9 were separated. (**Figure 4B**).

In comparison, only four different arrangements of chromosome 2 and 9 were observed in leaf G1 nuclei. Nuclei containing two nucleoli were not present. 36.7% of leaf nuclei contained two large CTs corresponding to chromosomes 2 and 9, other nuclei contained one large CT of chromosome 9 and two separated CTs specific to chromosome 2 (26.4%). In the similar number of nuclei (22.2%) CTs of homologous chromosome 2 were associated, and CTs of chromosome 9 were separated. Finally, 14.4% of leaf G1 nuclei contained both chromosomes arranged in separate CTs (**Figure 4B**).

The difference in proportions of separated and associated CTs between chromosomes 2 and 9 can be attributed to the presence of NOR region on the short arm of chromosome 9. NOR region consists of 45S rRNA genes which constitute nucleoli. Therefore, the position of chromosome 9 in interphase nuclei also depends on the position and nature of nucleolus/nucleoli (**Supplementary Figure 2**). A detailed 3D analysis revealed different numbers of 45S rDNA loci in the root and leaf G1 nuclei. In the root, two major loci were usually observed on the periphery of the nucleolus, with 2–4 small signals observed inside the nucleolus (**Supplementary Figure 2**; **Table 3**). In comparison, only 1–2 45S rDNA loci situated on the periphery of the nucleolus were observed in the leaf, where nucleolus occupies much lower volume (**Supplementary Figure 2**; **Table 3**).

**Table 3:** Analysis of G1 nuclei and 45S rDNA.

	Number of analyzed G1 nuclei	Mean volume of nucleus [µm3]	Mean volume of nucleolus [µm3]	Mean volume of 45S rDNA [µm3]	Mean number of 45S rDNA loci	Maximal number of 45s rDNA loci
**Root**	94	199.35 ± 45.76 100%	14.41 ± 4.56 7.23%	2.51 ± 1.16 1.26%	3.53 ± 1.49	6
**Leaf**	95	58.76 ± 20.26 100%	0.86 ± 0.18 1.47%	0.93 ± 0.31 1.58%	1.54 ± 0.50	2

**Figure 5:**
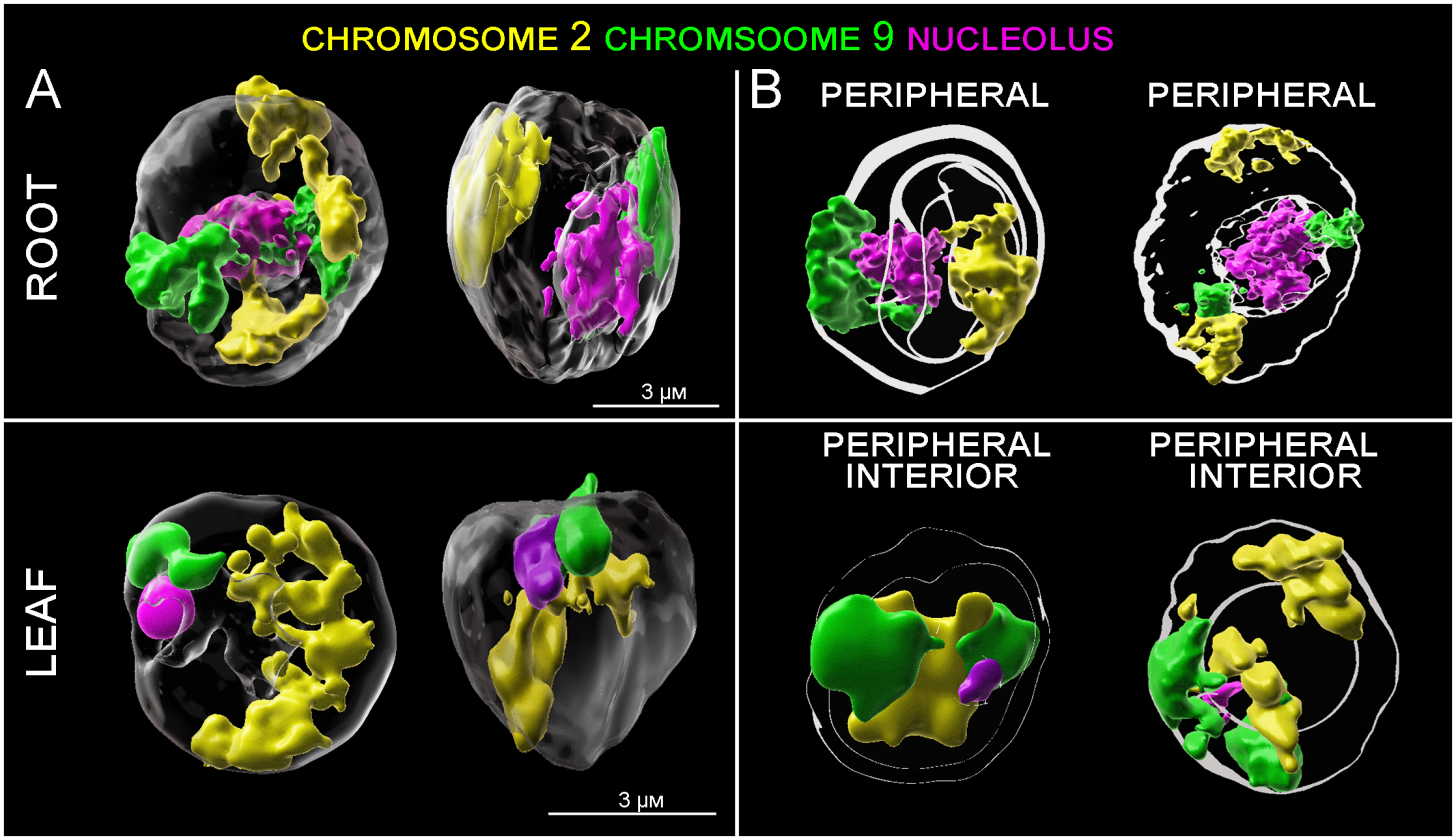
3D models of CTs positioning in root and leaf G1 nuclei. **(A)** Spatial positioning of CTs specific to chromosome 2 (yellow) and 9 (green) and nucleoli (red). **(B)** Model showing spatial arrangement of the CTs and nucleoli with respect to the center and periphery of the nucleus. Shells of equal area depict regions of the nuclei, where signals of DAPI (white), and chromosome 2 (yellow) and chromosome 9 (green) were localized.

Detailed image analysis of leaf G1 nuclei revealed that the position of chromosome 2 is more random, compared to chromosome 9. Large sub-metacentric chromosome 2 was, in most cases, arranged through the entire nucleus volume in the z-axis, with a large region located on the nuclear periphery (**Figure 5**). Although chromosome 2 does not contain rRNA genes and is not directly connected to nucleoli, its spatial positioning seems to be influenced by the nature of nucleoli (size, number, and position inside the nucleus).

As we mentioned above, the G1 nuclei of both tissues varied also in their shape (**Table 1**; **Figure 2B**), thus, we investigated the relations between the CTs arrangement and nuclei shapes. Our data showed, that specific arrangements of CTs did not correlate with different shapes of nuclei. Despite the lower proportion of spindle and flattened nuclei (**Table 1**; **Supplementary Figure 3**), all CTs rearrangements (two separated territories, and homologous chromosome associated territory specific to chromosomes 2 and 9) were present in all examined nuclei (**Supplementary Figure 3**). Nevertheless, the potential connection between the dominant pattern of CTs and the nuclear shape needs to be investigated in more detail on larger sample set due to unequal representation of spindle and flattened nuclei (**Table 1**; **Figure 2B**).

**Figure 6:**
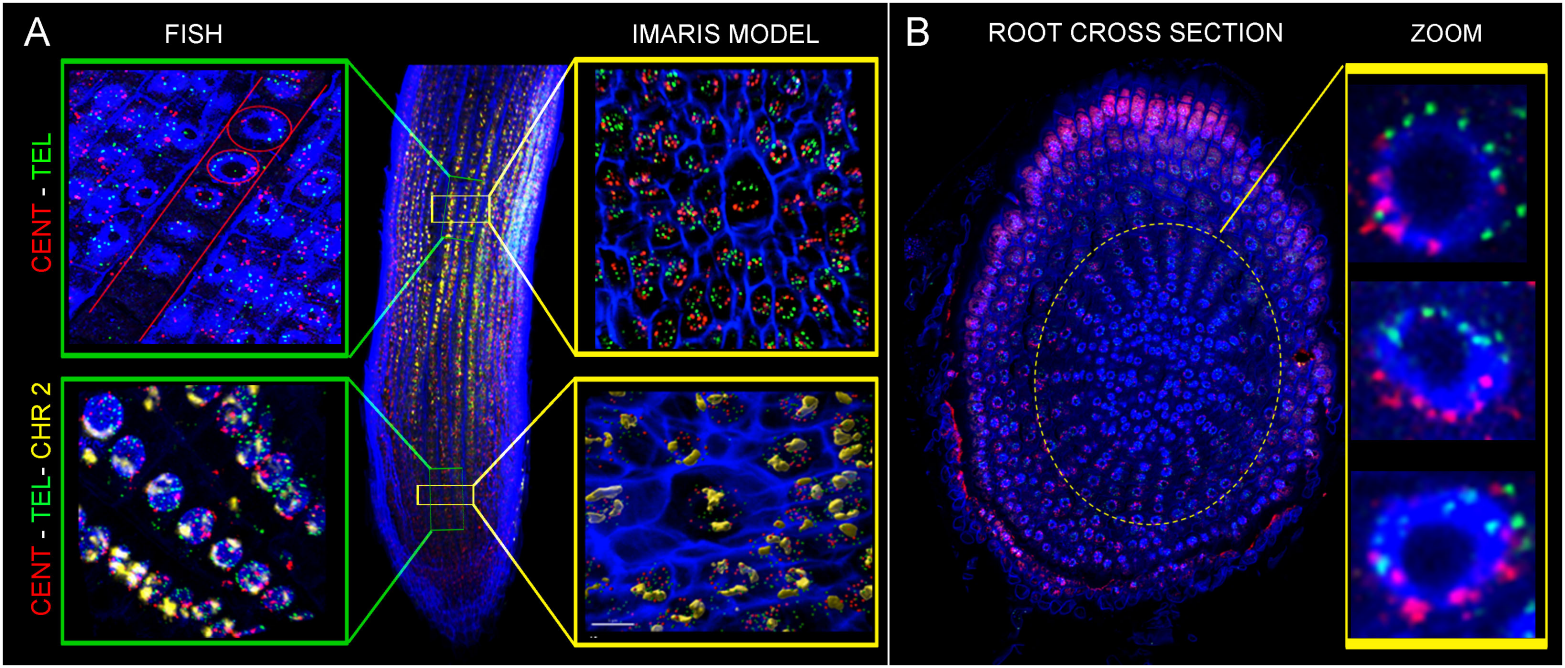
Oligo-painting FISH on root ultra-thin sections prepared by cryomicrotome. Centromeric probe (red), telomeric probe (green) and specific probe for chromosome 2 (yellow) were applied. Images displayed the evidence of Rabl configuration in xylem **(A)** and cortex **(B)** cells.

Finally, we localized probes specific to centromeric and telomeric sequences on ultra-thin root sections prepared by cryomicrotome. Rabl configuration was observed in rice xylem vessel cells, as well as in cortex cells (**Figures 6A, B**). Both cell types are larger, and the volume measurement of their nuclei performed in Imaris software indicates the presence of the endoreduplication. Unfortunately, the proportion of these specific cell types in roots of rice is very low, making it impossible to identify them by flow cytometry to estimate their DNA content, and confirm the presence of endoreduplication.

## Discussion

Early studies of chromatin structure using electron microscopy suggested its helical arrangement into 30 nm nucleosome fiber (Finch and Klug, 1976; Woodcock et al., 1984; Bordas et al., 1986). However, this model of chromatin folding and its higher-order organization became controversial due to the difference in observation between *in vivo* and *in vitro* conditions (Maeshima et al., 2019; Prieto and Maeshima, 2019). Recent development of super-resolution microscopy techniques, which can achieve a resolution of about 1–250 nm (reviewed in Valli et al., 2021), allowed to describe a presence of 100–200 nm higher-order chromonema fibers (Kireeva et al., 2004; Belmont, 2014). Studies of DNA replication foci in human cells proposed a globular folding of chromatin with a diameter of about 110–150 nm (Jackson and Pombo, 1998; Albiez et al., 2006; Cseresnyes et al., 2009; Markaki et al., 2012).

In our study, we have analyzed the chromatin compaction in the interphase nuclei of highly dynamic root meristematic cells and nuclei isolated from differentiated leaf cells. A striking difference in chromatin compaction in G1 nuclei of root and leaf tissues were observed (**Figure 1**). The presence of 72 nm chromatin fibers was revealed in rice G1 nuclei isolated from root meristem and young leaf tissue. Similar width of chromatin fiber (70 nm) was observed in metaphase chromosomes of Drosophila (Matsuda et al., 2010) and recently, in mitotic chromosomes of barley root meristem (Kubalová et al., 2023). These results could indicate that the higher level of chromatin spiralization, which is typical for mitotic chromosomes, is maintained in interphase nuclei of highly dynamic meristematic cells. On the contrary, more dense chromatin regions reaching 252 nm were observed in rice leaf nuclei. Similar variability in chromatin fibers was observed in human and animal studies, including metazoans (reviewed by Hansen et al., 2018). Studies of Belmont and Bruce (1994) and Dehghani et al. (2005) showed the presence of two classes of chromatin fibers, with diameters of 60–80 nm, and 100–130 nm in early G1 and late G1/early S. The described diameter of rice higher order chromatin structure correlates with the diameter of the He-la cells’ higher-order chromatin structure (220 nm) (Nozaki et al., 2017).

The root meristem and leaf nuclei varied also in the volume and level of chromatin compactness. Root meristem G1 nuclei were more than three times larger and consisted of more relaxed chromatin with higher proportion of interchromatin compartments (**Figure 1**). As we analyzed G1 nuclei of highly dynamic root meristem cells, we can speculate that the larger size of these nuclei and higher proportion of interchromatin compartments are needed for the synthesis of mRNA and proteins, which are required for DNA synthesis in the following S phase. The less compact chromatin of meristem root G1 nuclei can be also affected by the cell division dynamics in the root apical meristem (Kaduchová et al., 2023). The repeated and rapid division of the cells prevent the root meristem chromatin to be tightly condensed, and thus more flexible and accessible for the entire process of the cell division. In comparison, the differentiated cells of leaf tissues contained smaller G1 nuclei consisting of more compact chromatin with lower proportion of interchromatin compartments, where the transcription takes place, as was demonstrated in human studies (Hübner et al., 2015; Cremer and Cremer, 2019). We found, that chromatin of the root meristematic cells approximately two times more loosely packed compared to the leaf cells. This discrepancy in chromatin density may indicate different distances between the surface of arranged nucleosomes, as proposed previously by Gelléri et al. (2023).

Recently, it was shown that nuclear architecture, the size and shape, and positioning of CTs during interphase can be influenced by several factors, especially the size of a given chromosome, position of centromere, and the shape of nucleolus. In *Brachypodium distachyon*, a plant species that maintains Rabl configuration, a high level of homologous CTs associations was found in spherical nuclei, while it was negatively correlated with elongated nuclei (Robaszkiewicz et al., 2016). Similar results were described for plants with rosette-like chromosome conformation in the nuclei of both root and leaf tissues (Pečinka et al., 2004). However, our study did not confirm the correlation between the mutual position of two morphologically different chromosomes and the nuclear shape (**Supplementary Figure 3**). On the other hand, we showed differences in organization and mutual chromosome position between root meristem and leaf G1 nuclei. In root meristem nuclei, the CTs of NOR bearing chromosome 9 were mostly separated, while their association prevailed in leaf G1 nuclei, regardless the shape of nuclei. Our findings are not in agreement with previous study in *Brachypodium*, where the predominant association of CTs containing NOR region was demonstrated (Robaszkiewicz et al., 2016). Robaszkiewicz et al. (2016) also suggested, that the length of a particular chromosome may influence the dominant pattern of its spatial arrangement inside the nucleus, and showed that CTs of the longest chromosome were usually associated. However, the high level of variability in mutual chromosome organization, showed in the study of Robaszkiewicz et al. (2016), could be caused by the analysis of nuclei isolated from the pooled root tissue. Random positioning of most CTs was observed in *Arabidopsis*. The only exception was the position of NOR bearing chromosomes, which seemed to be connected to the position of nucleoli (Lysák et al., 2001; Pečinka et al., 2004; Berr and Schubert, 2007).

Spatial organization and mutual position of CTs in 3D space of large plant genomes with Rabl configuration have not yet been analyzed by *in situ* techniques. The only exception was the visualization of alien chromosomes in wheat-rye and wheat-barley introgression lines (Abranches et al., 1998; Koláčková et al., 2019; Perničková et al., 2019). In both cases, a complete separation of CTs corresponding to alien chromosomes was observed in majority (83 – 89%) of studied root meristem cells (Koláčková et al., 2019; Perničková et al., 2019). The discrepancies in CTs organization and positioning in 3D nuclear space between our work and previous studies, especially those of Robaszkiewicz et al. (2016), could be explained by the difference in chromosome configuration (Rabl and non-Rabl) in the studied species.

As we already mentioned, the shape and number of nucleoli represent another factor, which can affect the CTs positioning. Derenzini et al. (1998) showed, that cancer dividing cells produced elevated amounts of rRNA and often possessed large nucleoli, whereas down-regulation of rRNA gene transcription led to the reduction in nucleolar size. More recently, Tiku and Antebi (2018) showed, that the size of the nucleolus positively correlates with rRNA synthesis. Larger nucleoli volumes together with larger cumulative volume of 45S rDNA loci in the root cells (**Supplementary Figure 2**; **Tables 1**, **3**) revealed in our study, therefore indicate higher activity of rRNA genes in the root merisitematic cells compared to leaf cells. Homologs of chromosome 9 were organized into separated territories (in 93% of all events) in G1 nuclei of root meristem, where the rRNA genes are being highly expressed (Tulpová et al., 2022). On the other hand, CTs of chromosome 9 were more associated (59%) in leaf G1 nuclei, which consist of smaller nucleolus and few clusters of 45s rDNA. High rate of variability in mutual chromosome positioning in the 3D space of G1 nuclei isolated from both plant tissues, showed in our study, may reflects the interphase chromatin dynamics/movements. Movement of chromatin was described in *Arabidopsis* interphase nuclei by visualization of tagged loci in live seedlings (Kato and Lam, 2003), yeast (e.g. Heun et al., 2001; Bystricky et al., 2004; Hajjoul et al., 2013), and animal and human cells (e.g. Chubb et al., 2002; Levi et al., 2005; Germier et al., 2017; Nozaki et al., 2023).

The observed heterogeneity in chromosome positioning and variability in chromatin condensation within different tissues explain the discrepancy between the contact frequencies and the distance distributions obtained by Hi-C and 3D-FISH (Fudenberg and Imakaev, 2017). In plants, most Hi-C studies, which can be also used to create putative models of chromatin condensation and chromosome positioning, were done on pooled tissues (Wang et al., 2015; Dong et al., 2017; Concia et al., 2020). Therefore, 3D modeling was performed based on averages of large numbers of cells, and the information on potential variability in 3D structure among different cells or cell types was lost. This can be overcome by single-cell Hi-C (scHi-C) experiments (Nagano et al., 2013; Ramani et al., 2017; Tan et al., 2018). However, in plant research, scHi-C experiments are not numerous. For instance, in rice, this technique was used to study the variability in chromatin organization in eggs, sperm cells, unicellular zygotes, and shoot mesophyll cells. Even though the analysis was performed only on four cells representing each tissue type, theoretical models of chromosome folding and their mutual organization indicated the variability in the positioning of chromosome territories among the analyzed nuclei (Zhou et al., 2019).

To conclude, our study highlights the power of advanced microscopy combined with recent cytogenetics techniques to analyze and compare mutual chromosomes positions in the nuclei during the interphase of the cell cycle. Our experiments support the hypothesis, that chromatin organization is not determined by the shape of the nucleus. On the other hand, it appears that the size of nucleolus/nucleoli and their position in nucleus influence the chromosome positioning during interphase. The analysis of large number of nuclei confirms the variability in chromosome organization into nuclear territories and their mutual positioning within and also between the nuclei isolated from different tissue types. Furthermore, the use of super-resolution STED microscopy corroborates striking differences in chromatin folding and organization in the interphase nuclei isolated from the two studied plant tissues.

## Data availability statement

Publicly available datasets were analyzed in this study. This data can be found here: http://rice.uga.edu/.

## Author contributions

AD: Conceptualization, Investigation, Methodology, Writing – original draft, Writing – review & editing. DB: Investigation, Methodology, Writing – review & editing. VK: Methodology, Writing – review & editing. EH: Conceptualization, Investigation, Methodology, Supervision, Writing – original draft, Writing – review & editing.

## Supplementary material

Supplementary Figure 1 Example of chromatin measurements in root **(A)** and in leaf nuclei **(B)** in G1 phase. Nuclear DNA was counterstained by spirochrome (red).

Supplementary Figure 2 Differences in chromosome 9 arrangement (green) and 45S rDNA (yellow) activity in root and leaf. Models of individual arrangements were created based on raw data observation, using BioRender.com.

Supplementary Figure 3 Correlation between nucleus shape and CTs association.

Supplementary Video 1 Rice root nucleus in G1 phase. Chromosome 2 (yellow) and chromosome 9 (green) were visualized using oligo-painting FISH. Nucleolus was stained with immunolabeling with fibrillarin (red). Nuclear DNA was counterstained with DAPI (blue).

Supplementary Video 2 Rice leaf nucleus in G1 phase. Chromosome 2 (yellow) and chromosome 9 (green) were visualized using oligo-painting FISH. Nucleolus was stained with immunolabeling fibrillarin (red). Nuclear DNA was counterstained with DAPI (blue).
