# Supplementary figures and images for: Insight into chromatin compaction and spatial organization in rice interphase nuclei
Source: Front Plant Sci. 2024 May 28;15:1358760. doi: 10.3389/fpls.2024.1358760 (PMC11165205; doi:10.3389/fpls.2024.1358760)

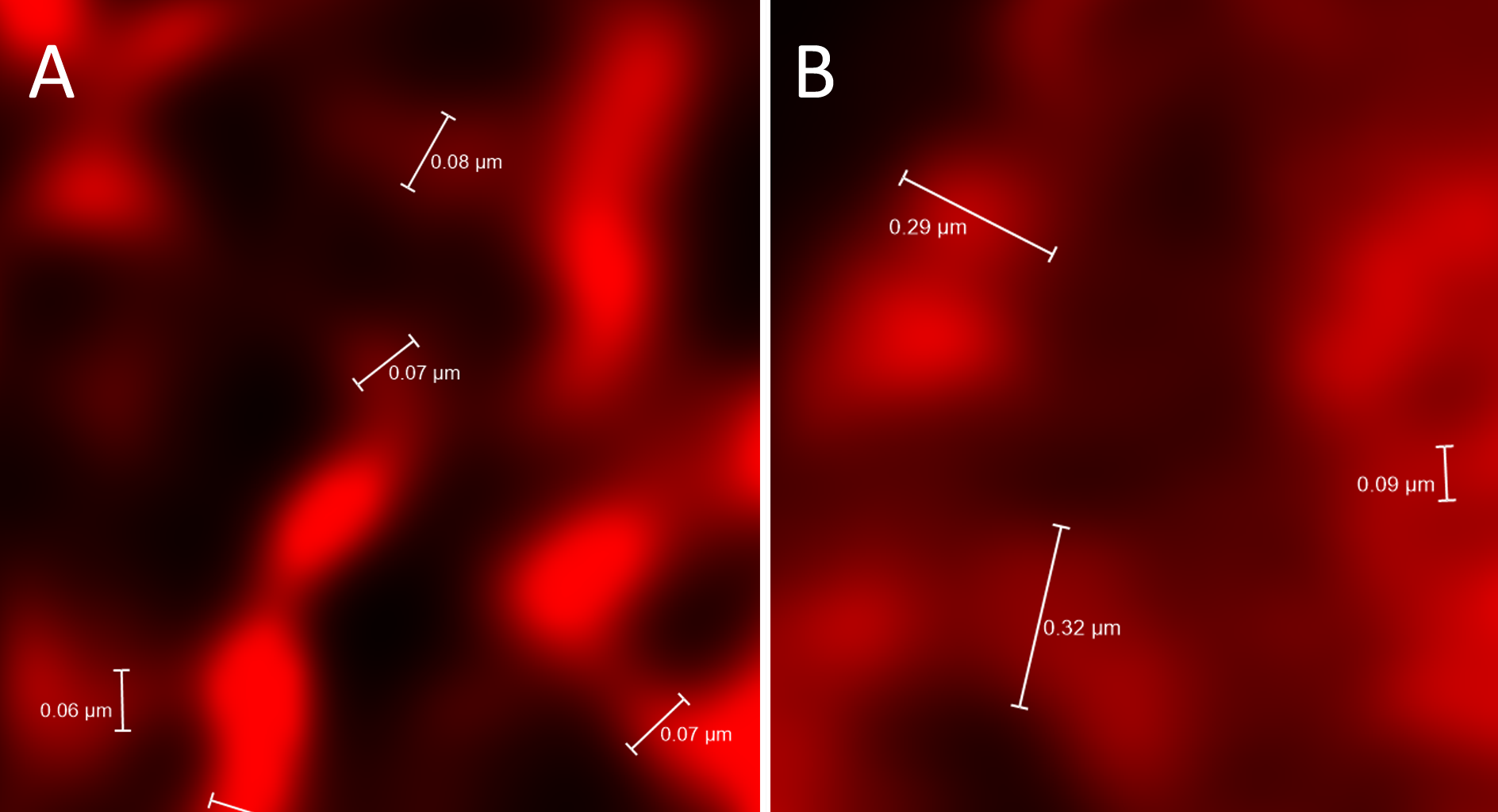

Supplement: Supplementary Figure 1 — Example of chromatin measurements in root (A) and in leaf nuclei (B) in G1 phase. Nuclear DNA was counterstained by spirochrome (red). [file Image_1.tif]

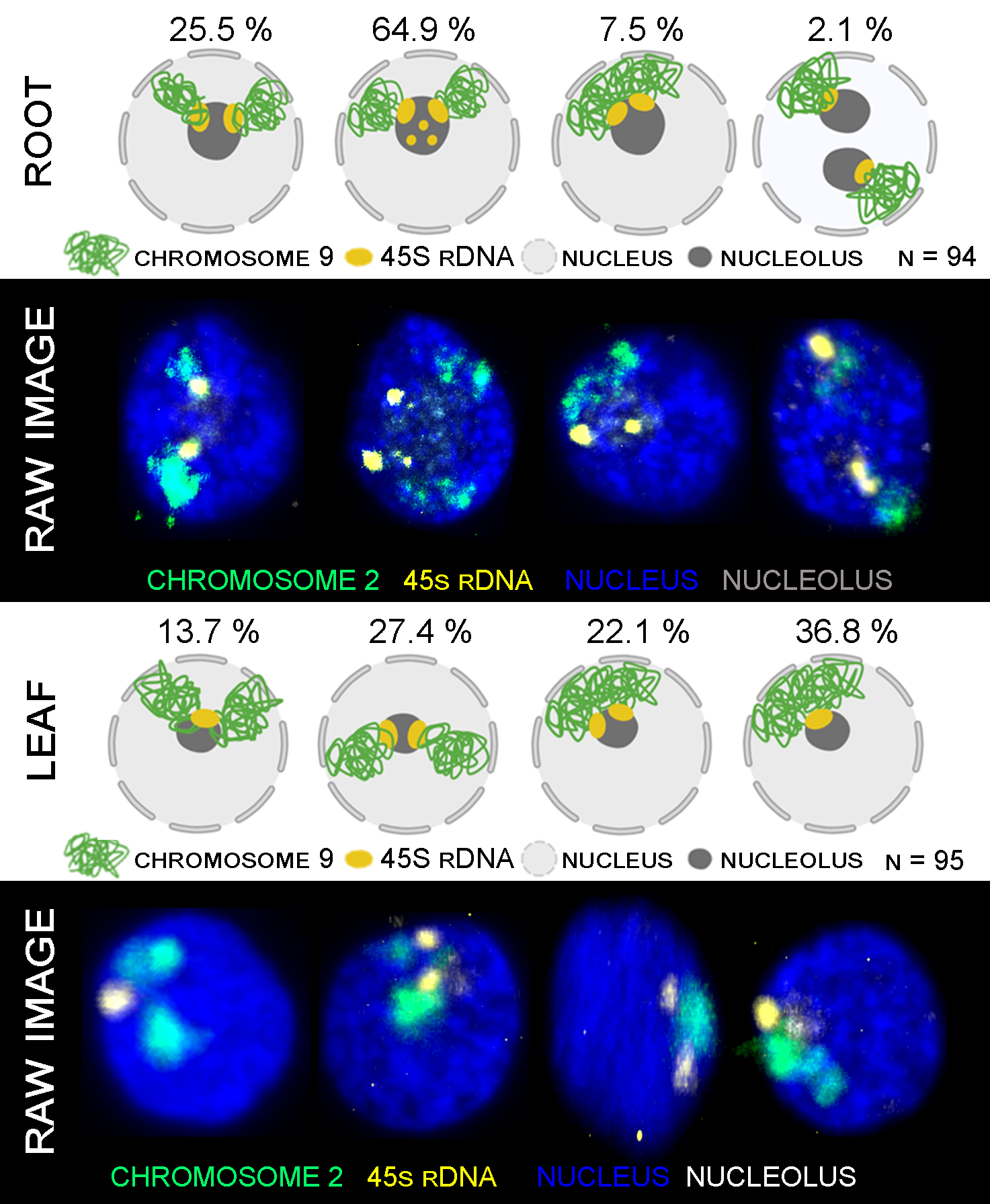

Supplement: Supplementary Figure 2 — Differences in chromosome 9 arrangement (green) and 45S rDNA (yellow) activity in root and leaf. Models of individual arrangements were created based on raw data observation, using BioRender.com. [file Image_2.tif]

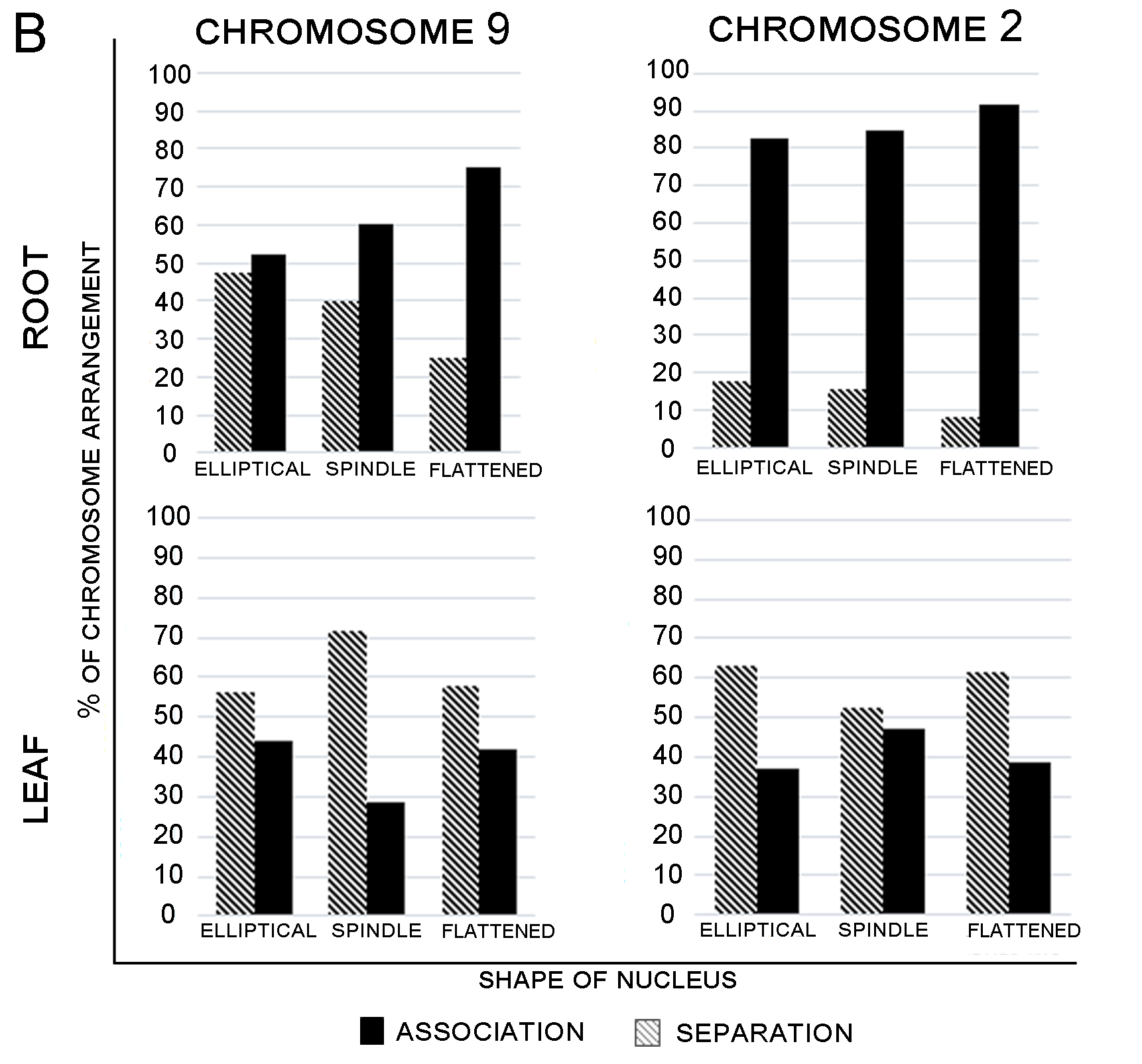

Supplement: Supplementary Figure 3 — Correlation between nucleus shape and CTs association. [file Image_3.tif]
